# Comparative anthropomorphism and affective valence shape role perception and relational behaviours in human-pet dyads in Romania

**DOI:** 10.3389/fpsyg.2026.1771900

**Published:** 2026-03-10

**Authors:** Florina Ileana Hica, Alina Simona Rusu, Iulia Francesca Pop, Ionel Papuc

**Affiliations:** 1 Department of Preclinical Sciences, Faculty of Veterinary Medicine, University of Agricultural Sciences and Veterinary Medicine, Cluj-Napoca, Romania; 2 Human-Animal Interaction Research Lab, Department of Fundamental Sciences, Faculty of Animal Science and Biotechnologies, University of Agricultural Sciences and Veterinary Medicine, Cluj-Napoca, Romania

**Keywords:** anthropomorphic valence, anthropomorphism, attitudes toward animals, companion animals, human-animal interaction, pet role, social role

## Abstract

This study examines the association between attitudes toward animals, anthropomorphic tendencies, and beliefs about the social role of companion animals (cats and dogs) in the household. We hypothesised that beliefs about the social role of companion animals are shaped both by general attitudes toward animals, including opinions about the emotional and cognitive abilities of the animals, and by a higher tendency to anthropomorphise expressed by pet owners. We investigated behavioural expressions, such as communication and reconciliation with the animal, and perceived social support as relational outcomes between owners and their pets. Data were collected via questionnaires from a sample of 445 cat and dog guardians from Romania, where cultural norms around companion animals are under-researched. The findings indicate that anthropomorphic thinking is a stronger predictor of the perceived social role of the pet in the familial structure than general attitudes toward animals. Moreover, our findings indicate that pet role perception partially mediates the relational outcomes: participants who ascribed their pets a more influential social role reported a higher level of communicative and reconciliatory behaviour, as well as greater social support. This study also explores the valence of anthropomorphic attribution, whether positive, negative, or mixed. The findings reflect that participants vary in the attribution of exclusively positive, exclusively negative, or mixed qualities to their pets. These results highlight nuanced psychological processes that are shaping the emotional and behavioural landscape of human-pet relationships, and can be further integrated in educational contexts, including preparation for adoption, fostering and pet management programs.

## 1 Introduction

The relationship between humans and non-human animals is one of the most enduring and complex social phenomena, shaped by both evolutionary history and contemporary cultural values. The importance of personal and societal attitudes and beliefs towards animals cannot be understated, as attitudes towards animals may act as determining factors of human behaviour and mediators of the valence (positive or negative) of the interactions that inform the quality of human and animal life (Rusu et al., 2017; Kisley, 2025). Consequently, changes in attitudes towards animals can drive legislative reform, animal rights advocacy, and education programs promoting animal welfare (Serpell, 2004; Apostol et al., 2013).

In the literature of human-animal interaction studies one of the most influential frameworks conceptualises attitudes toward animals as comprising both affective and utilitarian dimensions (Serpell, 2004). The affective dimension reflects empathy and moral concern, whereas the utilitarian dimension captures instrumental or functional evaluations of animals. Empirical work has consistently shown that these orientations are socially patterned, with women and younger individuals generally reporting more positive attitudes toward animals (Herzog et al., 1991; Apostol et al., 2013; Clark et al., 2016; Randler et al., 2021), and higher levels of education and income often associated with stronger welfare-oriented views (Clark et al., 2016; Su and Martens, 2018), although findings regarding religiosity remain mixed (Phillips et al., 2012; Su and Martens, 2018).

At the same time, cross-cultural research suggests that while the expression of these attitudes varies across socio-historical contexts, their underlying psychological structure may be relatively stable (Amiot and Bastian, 2015). For example, although Romanian respondents have demonstrated somewhat stronger utilitarian tendencies than participants in other cultural settings, affective orientations toward animals appear comparable across samples (Rusu et al., 2018). Such findings suggest that while moral frameworks are culturally embedded, the broader evaluative structure of attitudes toward animals may be relatively stable.

Building on these evaluative orientations, a key cognitive process through which attitudes toward animals are translated into everyday interaction is anthropomorphism, defined as the attribution of human-like emotions, thoughts, or motivations to non-human entities (Epley et al., 2008). While this may not be functionally wrong, the human-focused lens through which humans anthropomorphise entails using human moral constructs to describe non-human behaviours or inanimate objects (Horowitz and Bekoff, 2007), which may potentially lead to misinterpretations or misrepresentations of animal behaviour (Karlsson, 2012). In applied contexts, such distortions may have unintended welfare consequences. For example, higher anthropomorphic tendencies have been associated with pet obesity, suggesting that treating animals according to human dietary or nurturing norms may result in maladaptive care (Cao, 2014). More broadly, anthropocentric interpretations may reinforce dominion-based perspectives by filtering animal behaviour exclusively through human expectations (Blouin, 2012).

By contrast, Bekoff (2000) proposed the concept of biocentric anthropomorphism, which legitimises the use of anthropomorphic language when it serves to recognise animal emotions, provided interpretation remains anchored in the animal’s perspective.

Companion animals embody what Albert and Bulcroft (1988) termed an “economic paradox”: they hold emotional rather than functional value yet are often maintained with significant resources. The act of keeping pets inherently involves a degree of anthropomorphism or “personification” (Paul et al., 2014). Many owners name their pets, talk to them, take their photographs, and mourn them as they would human companions (Antonacopoulos and Pychyl, 2010; Kubinyi et al., 2009). The expansion of markets for pet luxury goods and services further illustrates how anthropomorphic meaning is socially and economically reinforced.

Anthropomorphic reasoning also varies across species and contexts. For example, Airenti (2018) notes that individuals may display inconsistent anthropomorphic tendencies, viewing companion animals as quasi-human while simultaneously perceiving farm animals as property or commodities. These contradictory opinions reveal how social relationships with animals (rather than abstract cognition) shape perceived human-likeness.

This relational perspective is particularly relevant in contexts such as Romania, where companion-animal practices have shifted in recent decades from predominantly utilitarian roles toward increasingly affect-oriented forms of pet keeping. Within such environments, anthropomorphising companion animals may function as a mechanism through which animals are recognised as sentient social partners, potentially expanding moral consideration and supporting welfare-oriented engagement. At the same time, its implications depend on how anthropomorphic interpretation is affectively framed and enacted within the owner-pet relationship.

In line with this relational framing, anthropomorphism can act as a bridge between human emotion and animal welfare, enhancing moral consideration and empathy (Epley et al., 2008; Serpell, 2002). Individuals who attribute rich emotional lives to animals are often more supportive of animal rights and ethical treatment (Brown and McLean, 2015). In this sense, anthropomorphic thinking can function as a communicative tool for creating compassionate interactions concerning not only human emotional needs, but also the perceived needs and welfare of companion animals (Kieson, 2025). Conversely, anthropomorphism does not occur only in magnitude but also in tone. Emotional attributions can be positively valenced (linked to nurturing, praise, and caregiving expectations) or negatively valenced, reflecting frustration or reproach, sometimes translated into aggressive behaviours or abandonment, when the animal’s behaviour conflicts with perceived norms (Morris et al., 2012). Such emotional polarity may shape how individuals interpret pets’ actions and assign social roles within the household (Airenti, 2018). Positively valenced anthropomorphism has been associated with attachment and supportive caregiving behaviours (Antonacopoulos and Pychyl, 2010; Paul et al., 2014), whereas negative attributions may diminish moral consideration or justify undesirable treatment (Serpell, 2004). Yet, despite its conceptual relevance, anthropomorphic valence has received limited empirical attention, with research largely focused on the degree of anthropomorphising rather than its affective direction. Understanding anthropomorphic valence as an affective polarity may help to explain why some pet owners engage in more nurturing and communicative behaviours, whereas others respond with frustration or withdrawal.

Within the household, these perceptions and attitudes converge in beliefs about the pet’s social role. Pets may be seen as children, friends, or simply as animals, reflecting variations in the beliefs about pet role (Albert and Bulcroft, 1988; Doré et al., 2019). Such role attributions are shaped by relational context and personal expectations, influencing the degree of emotional significance assigned to the animal. These roles are not merely symbolic; they correspond to measurable relational processes, including attachment-related dynamics observed in human-dog interactions (e.g., oxytocin-mediated bonding; Nagasawa et al., 2015), further underscoring the depth of these interspecies ties.

If pets are integrated into the family system through meaningful social roles, these role attributions should be reflected in observable relational outcomes. The role assigned to a pet can influence affiliative behaviours, conflict resolution, and perceived social support. Communication behaviour, both verbal (e.g., pet-directed speech) and non-verbal (e.g., petting, hugging), represents a core element of the human-animal bond (Airenti, 2018; Horowitz and Bekoff, 2007). Pets often become recipients of personal disclosure or “confidants” (for example, Veevers, 1985), illustrating how anthropomorphic tendencies underpin communication. Similarly, making-up behaviour (owners’ attempts to reconcile after perceived transgressions) reflects the application of human moral scripts to interspecies relationships (Cryder et al., 2012; Kogan et al., 2022). Guilt and empathy are considered to drive these reparative gestures, which may benefit both the owner’s emotional state and the pet’s welfare.

Beyond behavioural expressions, pets often serve as alternative or complementary sources of social support. They are seen as providers of emotional stability, affection, and a sense of acceptance (Franklin, 1999, as cited in Irvine and Cilia, 2017; Hill et al., 2020). Some studies suggest that pets can compensate for limited human social support (Veevers, 1985; Antonacopoulos and Pychyl, 2010), while others argue that they enhance existing social bonds (McNicholas et al., 2005; Paul et al., 2014). Anthropomorphic interpretation plays a central role here: owners who perceive their pets as emotionally responsive are more likely to experience them as supportive companions (Serpell, 2002; Antonacopoulos and Pychyl, 2010; Paul et al., 2014; Prato-Previde et al., 2022). Thus, social support can act both as a product of anthropomorphism and as a reinforcer of it.

Taken together, these perspectives reveal how attitudes towards animals, anthropomorphic tendencies, and beliefs about pets’ social roles interact to shape the emotional and behavioural dynamics of human-pet relationships. Previous research has robustly documented bi-dimensional attitudes (Serpell, 2004), demographic predictors (e.g., Herzog et al., 1991; Clark et al., 2016), cross-cultural variations (Rusu et al., 2018; Amiot and Bastian, 2015), and anthropomorphism’s dual potential to foster empathy or distort welfare (Epley et al., 2008; Bekoff, 2000; Cao, 2014). Studies have also linked these factors to relational outcomes like communication, attachment, and social support, often highlighting anthropomorphism’s role in role attribution (Airenti, 2018; Doré et al., 2019; Antonacopoulos and Pychyl, 2010). However, critical gaps persist: most work focuses on the *degree* of anthropomorphism rather than its *affective valence*, the positive (nurturing) versus negative (frustrated) emotional tone of attributions, which remains empirically underexamined despite its theorized influence on caregiving and conflict (Morris et al., 2012; Serpell, 2004). Moreover, few studies test *integrated models* where attitudes and anthropomorphism predict outcomes *via mediation* by pet role perceptions, particularly in non-Western contexts.

This scarcity of research is especially pronounced in Central and Eastern Europe, including Romania, where empirical research has largely examined attitudes or attachment patterns in isolation rather than testing integrated relational models. Although Romania represents a context characterised by rapid shifts in companion-animal practices and evolving welfare norms (Rusu et al., 2018; Blouin, 2012), few studies have examined how attitudes, comparative anthropomorphism, and affective valence jointly structure relational outcomes through pet role perception.

Viewed through the lens of the recently developed Interspecies Relational Theory (Kieson, 2025), these findings and gaps underscore the need to conceptualize human-pet relationships as relational systems shaped by meaning-making, affective orientation, and role attribution. IRT posits that attitudes toward animals and anthropomorphic interpretations influence relational outcomes not directly, but through the roles humans assign to animals and the emotional tone accompanying these interpretations. From this perspective, anthropomorphism is not inherently beneficial or harmful; rather, its function depends on how it is cognitively framed and affectively enacted within the relationship.

Although attitudes toward animals and anthropomorphic tendencies have been extensively documented, their joint operation within an integrated relational system remains insufficiently specified. In particular, limited empirical work has examined how these constructs translate into everyday interaction patterns through the social roles assigned to companion animals. Building on this gap, the present study tests an integrated relational model in which pet role perception functions as a central mediating mechanism linking attitudes toward animals, comparative anthropomorphism, and anthropomorphic valence to key relational outcomes. In addition, the study introduces anthropomorphic valence as an exploratory construct to assess whether the emotional tone of anthropomorphic attribution further differentiates relational patterns. Situating this integrated model within the Romanian cultural context responds to IRT’s call for context-sensitive analyses of interspecies relationships and extends existing research beyond Western-dominated samples.

Based on this framework, we hypothesised that more positive attitudes toward animals and stronger comparative anthropomorphic tendencies would be associated with greater pet role perception. We further expected pet role perception to mediate the relationships between attitudes and anthropomorphism, on the one hand, and relational outcomes (communication behaviour, making-up behaviour, and perceived social support), on the other. Finally, we anticipated that positively valenced anthropomorphic attributions would be associated with stronger relational bonds and more affiliative behavioural engagement toward the pet.

## 2 Materials and methods

### 2.1 Study design and context

This quantitative, cross-sectional, correlational study was inspired by the work of Bouma et al. (2023), who investigated how anthropomorphism and pet role perception predict relational outcomes in companion animal guardians and their pets. In line with the conceptual model proposed by Bouma et al. (2023), the present study employed their newly developed Comparative Anthropomorphisation Questionnaire (COANT), together with the pet role perception, communication behaviour, making-up behaviour, and social support items adapted in their study from earlier scales on social bonding, guilt-related behaviour and its dissolution, and perceived support. Unlike Bouma et al. (2023), who contrasted COANT with the Individual Differences in Anthropomorphism Questionnaire (IDAQ; Waytz et al., 2010), we used the Animal Cognition and Feelings subscale of the Attitudes Toward Animals Questionnaire (Fehlbaum et al., 2010), as we considered it more suitable for capturing moral and affective orientations in the Romanian population. In addition, we explored anthropomorphic valence as an independent dimension of anthropomorphic attribution that is theoretically relevant to owner-pet relationships.

This adaptation ensured both theoretical continuity with Bouma et al. (2023) and cultural and conceptual relevance to the local research population, while maintaining psychometric comparability across constructs central to the study of pet-owner relationships.

### 2.2 Participants and procedure

This study investigated the associations between attitudes towards animals, anthropomorphic tendencies, pet role perception, and relational outcomes among Romanian companion-animal guardians, also referred to as pet owners throughout the study. The target population consisted of adult dog and cat owners residing in Romania. Data were collected through an anonymous online questionnaire distributed via social media platforms and mailing lists over a period of 6 weeks beginning in November 2024.

Participants were required to be at least 18 years old, Romanian citizens residing in Romania, and to currently have at least one dog or cat in their household. Of the 493 respondents who completed the questionnaire, 445 questionnaires provided valid and complete data and were retained for analysis. The sample included both dog owners (*n* = 279) and cat owners (*n* = 166). Ages ranged from 18 to over 60 years (M = 32.4, SD = 11.6), with a predominance of female respondents (approximately 84%).

Participation was voluntary, anonymous, and without compensation, monetary or otherwise. Before accessing the questionnaire, participants provided informed consent after reading a short description of the study’s aims and their rights regarding confidentiality and withdrawal. The research was approved by the Bioethics Committee of the University of Agricultural Sciences and Veterinary Medicine of Cluj-Napoca (approval number 499/27.03.2025). All procedures complied with national and institutional guidelines for research involving human participants.

### 2.3 Measures

Demographic information was collected using closed items with fixed response options covering gender, age group, highest level of education, religiosity, and professional involvement with animals. Pet ownership was assessed by identifying the species of the companion animal (only cats and dogs were included in the study) and requesting the pet’s name; following the procedure used by Bouma et al. (2023), the survey software subsequently embedded the pet’s name into all relevant items to personalise the questionnaire. Participants owning more than one companion animal were instructed to answer with reference to a single focal pet of their choice. Additional pet characteristics were gathered through open-ended items, including the pet’s age, duration of ownership, breed, and sex.

#### 2.3.1 Attitudes toward animals (ATA)

Attitudes toward animals were assessed using the Animal Cognition and Feelings subscale of the Attitudes Toward Animals Questionnaire (Fehlbaum et al., 2010), which measures perceptions of animals’ emotional experiences, cognitive abilities, and moral consideration. This subscale has been previously validated and used in Romanian samples, showing satisfactory internal consistency in earlier studies (*α* = 0.79; Rusu et al., 2017).

The subscale consists of seven items rated on a 5-point Likert scale (1 = “strongly disagree,” 5 = “strongly agree”). Two items serve as control statements assessing general comprehension (“Animals’ feelings are different from those of people”; “Animals cannot think”), while the remaining items address animal emotions (e.g., “Animals have feelings, for example fear, joy”), cognition (e.g., “Animals can think like people”), and moral considerations regarding pain and euthanasia (e.g., “If an animal is suffering and cannot be cured, it should be killed painlessly”). Higher scores reflect stronger endorsement of animals’ cognitive, emotional, and moral relevance.

Internal consistency of this subscale in the present sample was *α* = 0.378, which can be either a result of the low number of items or of the fact that the items were measuring more than one concept, such as the beliefs in animal emotional and cognitive abilities on one hand, and moral attitudes regarding animal treatment (e.g., euthanasia) on the other hand.

#### 2.3.2 Comparative anthropomorphisation (COANT)

Anthropomorphic tendencies were measured using the Comparative Anthropomorphisation Questionnaire (COANT), developed by Bouma et al. (2023) to assess perceived similarities between human and animal cognitive and emotional capacities. The scale has demonstrated excellent internal reliability in previous research (*α* = 0.95; Bouma et al., 2023) and has shown positive associations with broader anthropomorphism measures such as the IDAQ.

COANT consists of 28 items rated on an 8-point Likert-type scale ranging from 0 (“no”) to 7 (“exactly like humans”). The available response options with intermediate choices allow participants to indicate graded degrees of similarity (e.g., “slightly similar to humans,” “moderately similar,” “very similar”), enabling a nuanced assessment of how closely respondents perceive their pet’s emotional or cognitive experiences to resemble those of humans. Example items include: “Do you think ___ has the capacity to reason?,” “Do you think ___ has the capacity to judge?,” and “Do you think ___ has the capacity to feel jealousy?.” Higher scores indicate stronger comparative anthropomorphism.

As the instrument had not previously been used in Romanian contexts, the English version was translated and culturally adapted for the present study following standard translation and back-translation procedures.

Internal consistency in the present sample was *α* = 0.967, indicating an excellent reliability of the scale.

#### 2.3.3 Pet role perception (PRP)

Pet role perception was measured using the four-item scale adapted by Bouma et al. (2023), which assesses the extent to which owners regard their companion animals as emotionally significant members of the family. The scale evaluates roles ranging from child and friend to full family member or simply a pet. In previous research, this scale demonstrated acceptable internal consistency (*α* = 0.77; Bouma et al., 2023).

Items include: “___ is like a child to me,” “Love for animals is real love,” “___ is a full member of the family,” and “___ is a true friend.” Responses were given on a 7-point Likert scale ranging from 1 (“strongly disagree”) to 7 (“strongly agree”), with higher scores indicating stronger emotional integration of the pet in the family system.

Because no Romanian version of this instrument was available, the items were translated and culturally adapted following standard translation and back-translation procedures. Internal consistency in the present sample was *α* = 0.857, indicating very good reliability of the scale.

#### 2.3.4 Relational outcomes

Three relational outcomes were assessed: communication behaviour, making-up behaviour, and perceived social support. All items were rated on 7-point Likert scales, with higher scores indicating stronger endorsement of the respective behaviours or perceptions.

- Communication Behaviour (CB): Communication behaviour was measured using five items assessing verbal and non-verbal affiliative interactions with the pet (e.g., talking to the pet, petting, hugging). Response options ranged from 1 (“never”) to 7 (“very often”). Internal consistency in the present sample was *α* = 0.842.
- Making-Up Behaviour (MUB): Making-up behaviour was measured using six items that described reparative actions taken following perceived wrongdoing toward the pet (e.g., apologising, giving treats, providing extra attention). Responses ranged from 1 (“never”) to 7 (“very often”). Internal consistency in the present sample was *α* = 0.838.
- Perceived social support (SS) from the pet was assessed using nine items adapted from Bouma et al. (2023), who based their measure on established human social support scales. In contrast to their 10-item adaptation, one item was unintentionally omitted during questionnaire construction. Responses were rated on a scale from 1 (“strongly disagree”) to 7 (“strongly agree”), where higher scores indicated greater perceived emotional support from the pet. Internal consistency in the present sample was *α* = 0.937, indicating an excellent reliability.

#### 2.3.5 Anthropomorphic valence

Anthropomorphic valence was explored using two single-item measures capturing the emotional tone of human-like language used to refer to the pet. One item assessed the use of affectionate or endearing expressions, while the second asked about uncomplimentary or reproachful expressions commonly directed toward humans. The items were presented as:

Do you use endearing words or expressions (e.g., ‘my baby’, ‘mummy’s baby’, ‘daddy’s baby’, or ‘kiddo’) when referring to ___?

Do you use uncomplimentary or reproachful words or expressions (e.g., ‘jerk’ or ‘bastard’) when referring to ___?

Both items were rated on a 7-point Likert scale (1 = “never,” 7 = “always”), with higher values indicating more frequent use of the respective anthropomorphic descriptors. The two items were analysed separately as indicators of positively and negatively valenced anthropomorphic language. Additional exploratory analyses involving derived categorical variables are presented in the Results section.

### 2.4 Data handling and statistical analysis

All analyses were conducted in JASP (version 0.19.3; JASP Team, 2024). Analyses were conducted within an integrated framework designed to examine how general attitudes toward animals (ATA) and comparative anthropomorphism (COANT) relate to relational outcomes through pet role perception (PRP). Preliminary analyses (descriptives, group comparisons, correlations) were used to characterise the sample and establish associations among constructs. Subsequently, regression and mediation models tested PRP as a central relational mechanism linking attitudes and anthropomorphic attributions to behavioural and socio-emotional outcomes. Moderated mediation analyses examined whether these pathways differed by pet species. Finally, exploratory analyses of anthropomorphic valence were conducted to investigate affective differentiation within anthropomorphic attribution. Together, these analyses form a coherent analytical sequence aligned with the study’s conceptual framework.

Because all items required responses, the final dataset contained no missing values. Composite scores for each construct (ATA, COANT, PRP, CB, MUB, SS) were computed as the mean of their respective items.

Data distribution was examined using the Shapiro–Wilk test, which indicated deviations from normality for most subscales. Consequently, non-parametric methods were used when appropriate. Preliminary analyses involved Mann–Whitney *U* tests to compare cat and dog owners and Spearman correlations to assess relationships among demographic and psychological variables. Based on these analyses, age and gender were retained as covariates, whereas education level and professional involvement with animals were excluded from further models.

To test the primary hypotheses, multiple linear regression models were estimated with communication behaviour (CB), making-up behaviour (MUB), and perceived social support (SS) as dependent variables. Predictors were attitudes toward animals (ATA), comparative anthropomorphism (COANT), and pet role perception (PRP), controlling for age and gender. Regression assumptions (linearity, homoscedasticity, independence, normality of residuals, and multicollinearity) were checked using residual diagnostics, VIF, and the Durbin–Watson statistic, and were reasonably met.

To examine whether PRP mediated the associations between ATA/COANT and the three relational outcomes (CB, MUB, SS), bootstrapped mediation analyses were conducted using 5,000 resamples with bias-corrected 95% confidence intervals. The mediation logic follows prior work demonstrating the centrality of pet role perception in owner-pet relationships (Bouma et al., 2023). Mediation was considered significant when the confidence interval did not include zero. Moderated mediation models additionally tested whether these indirect effects differed between dog and cat owners. Although these analyses follow conditional process logic, they were performed using JASP’s path modelling interface rather than Hayes’ PROCESS macro and therefore do not correspond to a specific PROCESS model number.

Anthropomorphic valence was examined using two single-item indicators. Additional categorical analyses were computed to differentiate respondents who predominantly used positive versus negative valenced anthropomorphic expressions.

All tests were two-tailed, with statistical significance set at *p* < 0.05.

Generative artificial intelligence (ChatGPT-4, OpenAI) was used to assist with wording and rephrasing of the presentation of the statistical results. All statistical analyses and their interpretation were conducted solely by the authors, who take full responsibility for the results presented in this study.

### 2.5 Data availability

The datasets generated and analysed during the current study are available from the corresponding author on reasonable request.

## 3 Results

Results are presented following the logic of the proposed conceptual framework, moving from descriptive and associative analyses to predictive, mediational, and exploratory affective analyses.

### 3.1 Psychometric evaluation of the study measures

Analyses were conducted on six composite scales (ATA, COANT, PRP, CB, MUB, and SS). For each construct, composite scores were calculated as the mean of all its corresponding items to maintain scale comparability and, at the same time, preserve the conceptual properties of the original measures.

Internal consistency was evaluated using Cronbach’s alpha. The COANT (*α* = 0.97), PRP (*α* = 0.86), CB (*α* = 0.84), MUB (*α* = 0.84), and SS (*α* = 0.94) subscales demonstrated high to excellent reliability. In contrast, the ATA subscale demonstrated low internal consistency (*α* = 0.38), which is considerably lower than its previously reported reliability in Romanian samples (*α* = 0.79; Rusu et al., 2017). Therefore, ATA was retained as a theoretically relevant construct, but its results were interpreted with caution.

Following reliability assessment, data distributions were examined using the Shapiro–Wilk test. All composite variables significantly deviated from normality (*p* < 0.05), and consequently, non-parametric methods were used where appropriate in subsequent analyses.

Because these variables exhibited adequate reliability (except for ATA, reported with interpretive caution), subsequent analyses were conducted using the composite mean scores of each construct. To contextualise the psychological patterns observed, we first describe the demographic and pet-related characteristics of the respondents.

### 3.2 Participant and pet characteristics

A total of 445 Romanian companion-animal guardians were included in the study. The sample was predominantly female (81.4%), with male respondents comprising 18.6%. Participants’ ages were distributed across five categories: 17.8% were 19–29 years old, 32.1% were 30–39 years old, 23.4% were 40–49 years old, 8.8% were 50–59 years old, and 17.9% were aged 60 or older, indicating a broad adult sample spanning young to older adults.

The majority of respondents reported having completed higher education (87%), while 7.6% had finished high school, 4.3% reported technical or vocational training, and 1.1% had only elementary or middle-school education. The level of self-reported spirituality was widely distributed, with most respondents indicating either moderate levels (36.6%) or low to very low levels (36.9% combined), while a smaller proportion reported high to extremely high levels (26.3%). Only a small proportion worked professionally with animals (5.6%), suggesting that the sample represents typical pet owners, and not animal-industry professionals.

Participants reported either a dog or a cat as their focal pet, with dog owners forming the majority (*n* = 279; 62.7%). Companion animals showed wide age variation, which ranged from infancy to old age: cats averaged 6.75 years (SD = 4.44) and dogs 6.45 years (SD = 3.89). The mean duration of ownership was comparable between species, with cat owners reporting an average of 6.21 years (SD = 4.26) and dog owners 6.02 years (SD = 3.93). With respect to pet sex distribution, 59% of reported cats were female and 41% male, whereas dogs were more evenly represented (49.1% female, 50.9% male). These descriptors were collected to characterise the sample but are not used as predictors in subsequent analyses; instead, pet type (dog vs. cat) serves as the main grouping variable. See Tables 1, 2 for detailed characteristics of participants and pets.

**Table 1:** Nominal demographic variables of participants.

Variable	Category	Number (%)
Gender	Female	362 (81.4%)
	Male	83 (18.6%)
Age	19–29 years	79 (17.8%)
	30–39 years	143 (32.1%)
	40–49 years	104 (23.4%)
	50–59 years	39 (8.8%)
	over 60 years	80 (17.9%)
Education level	Elementary and middle school	5 (1.1%)
	High school	34 (7.6%)
	Non-university/vocational or technical school	19 (4.3%)
	Higher education	387 (87%)
Self-reported degree of spirituality	Extremely small	72 (16.2%)
	Very small	41 (9.2%)
	Small	52 (11.7)
	Moderate	163 (36.6)
	Big	77 (17.3)
	Very big	29 (6.5%)
	Extremely big	11 (2.5%)
Working professionally with animals	Yes	25 (5.6)
	No	420 (94.4)

**Table 2:** Descriptive statistics and sex distribution for pets.

Statistics	Cat age (years)	Dog age (years)	Time owned cat (years)	Time owned dog (years)	Cat sex = female	Cat sex = male	Dog sex = female	Dog sex = male
Total	166 (37.3%)	279 (62.7%)			98 (59%)	68 (41%)	137 (49.1%)	142 (50.9%)
Mean	6.75	6.45	6.21	6.02				
SD	4.44	3.89	4.26	3.93				
Minimum	0.33	0.33	0.33	0.33				
Maximum	20	16	20	16				

### 3.3 Pet owner comparisons

Differences between cat and dog owners were examined across demographic characteristics using chi-squared tests of independence. A small but significant association emerged between gender and type of pet owned, *χ*^2^(1) = 4.01, *p* = 0.045, Cramér’s *V* = 0.10, indicating that women were more likely to own cats than men. Age was also significantly associated with pet ownership, *χ*^2^(4) = 10.35, *p* = 0.035, Cramér’s *V* = 0.15. Younger adults (19–39 years old) were more likely to own dogs, whereas middle-aged and older adults showed a slightly greater tendency to own cats. In contrast, education level was not significantly related to pet type, *χ*^2^(3) = 2.96, *p* = 0.397, Cramér’s V = 0.08.

Pet characteristics were also compared between species. An independent-samples Welch *t*-test was used to compare pet age due to unequal variances (Levene’s test: *F*(1, 443) = 5.43, *p* = 0.020). Pet age did not differ significantly between cats and dogs, Cohen’s *d* = 0.07, indicating negligible practical differences. A similar pattern emerged for duration of ownership, with no meaningful between-group variation observed.

Overall, these preliminary results indicate that demographic characteristics (particularly gender and age), but not education or pet characteristics (age and ownership duration), differentiate cat and dog owners in this sample.

To evaluate whether species-specific ownership relates to relational attitudes and behaviours, subsequent analyses compared the key psychological and behavioural constructs across cat and dog owners.

### 3.4 Differences between cat and dog owners on psychological and relational constructs

Group comparisons on the key psychological and behavioural constructs were conducted using Mann–Whitney *U* tests. Dog owners scored significantly higher on COANT (*U* = 27,346, *p* = 0.001, *r* = 0.18) and on SS (*U* = 26,040, *p* = 0.028, *r* = 0.12) than cat owners. These small effects suggest modest but meaningful differences in how guardians interpret their pets’ emotional and cognitive capacities and in the degree of support they perceive from them. No significant differences emerged between cat and dog owners for ATA (*U* = 23,894, *p* = 0.573, *r* = 0.03), PRP (*U* = 24,813, *p* = 0.200, *r* = 0.07), CB (*U* = 22,938, *p* = 0.865, *r* = −0.01), or MUB (*U* = 24,722, *p* = 0.233, *r* = 0.07), indicating broad similarity between species groups in these domains. Full descriptive statistics for both groups, along with mean ranks and distribution indices, are presented in Table 3.

**Table 3:** Group descriptives across all sub-scales.

Variable	Group	N	Mean	SD	SE	Mean rank	Sum rank
ATA	Cat	166	3.79	0.43	0.03	218.56	36,281.50
	Dog	279	3.82	0.48	0.03	225.64	62,953.50
COANT	Cat	166	1.89	1.26	0.10	197.77	32,829.50
	Dog	279	2.43	1.59	0.10	238.01	66,405.50
PRP	Cat	166	5.99	1.10	0.09	213.03	35,362.50
	Dog	279	6.05	1.17	0.07	228.93	63,872.50
CB	Cat	166	5.92	1.27	0.10	224.32	37,237.50
	Dog	279	5.92	1.20	0.07	222.21	61,997.50
MUB	Cat	166	4.27	1.19	0.09	213.57	35,453.00
	Dog	279	4.41	1.28	0.08	228.61	63,782.00
SS	Cat	166	5.24	1.26	0.10	205.64	34,135.50
	Dog	279	5.49	1.21	0.07	233.33	65,099.50

ATA = attitudes toward animals; COANT = comparative anthropomorphism; PRP = pet role perception; CB = communication behaviour; MUB = making-up behaviour; SS = social support.

To determine whether demographic variables should be statistically controlled in subsequent analyses, Spearman rank correlations were computed between participant characteristics and all six psychological and behavioural measures. Gender showed significant associations with several outcomes, including ATA (*ρ* = 0.10, *p* = 0.042), PRP (*ρ* = 0.13, *p* = 0.006), CB (*ρ* = 0.21, *p* < 0.001), and MUB (*ρ* = 0.13, *p* = 0.005). Age was consistently and negatively associated with all sub-scales: ATA (*ρ* = −0.20), COANT (*ρ* = −0.30), PRP (*ρ* = −0.24), CB (*ρ* = −0.41), MUB (*ρ* = −0.34), and SS (*ρ* = −0.19), indicating that younger participants reported stronger relational attitudes and behaviours toward their pets.

In contrast, education level and working professionally with animals showed no meaningful associations with any psychological or behavioural measure (all *p* > 0.10). Therefore, gender and age were retained as covariates in subsequent regression and mediation models, whereas education and professional involvement with animals were not included further.

To better understand how the measured constructs relate to one another at the individual level, correlations were examined among all psychological and behavioural variables.

### 3.5 Correlations among the study variables

Spearman rank correlations revealed significant positive associations among all six constructs (Table 4). Higher levels of ATA and COANT were associated with stronger PRP as well as more frequent CB and MUB. Perceived social support (SS) showed particularly strong associations with PRP, CB, and MUB, indicating that relational behaviours co-occur with the emotional experience of support from the pet.

**Table 4:** Spearman’s correlations of research variables.

Variable	Statistic	ATA	COANT	PRP	CB	MUB	SS
ATA	Spearman’s rho	–					
	*p*-value	–					
COANT	Spearman’s rho	0.471***	–				
	*p*-value	<0.001	–				
PRP	Spearman’s rho	0.352***	0.420***	–			
	*p*-value	<0.001	<0.001	–			
CB	Spearman’s rho	0.289***	0.358***	0.599***	–		
	*p*-value	<0.001	<0.001	<0.001	–		
MUB	Spearman’s rho	0.291***	0.413***	0.578***	0.617***	–	
	*p*-value	<0.001	<0.001	<0.001	<0.001	–	
SS	Spearman’s rho	0.323***	0.452***	0.674***	0.540***	0.538***	–
	*p*-value	<0.001	<0.001	<0.001	<0.001	<0.001	–

*p < 0.05, **p < 0.01, ***p < 0.001.

Based on these correlations, regression models were estimated to determine which factors best predicted behavioural outcomes.

### 3.6 Multiple linear regressions predicting relational outcomes

To examine the direct effects of the psychological constructs on each relational outcome, a series of multiple linear regression analyses was conducted. In each model, one focal predictor (ATA, COANT, or PRP) was entered, along with the covariates of gender and age. This analytical strategy enabled the unique contribution of each construct to be evaluated while statistically accounting for demographic influences.

#### 3.6.1 Communication behaviour (CB)

In the first set, the ATA model positively predicted CB (*B* = 0.60, SE = 0.11, *β* = 0.23, *t* = 5.33, *p* < 0.001). Gender also significantly predicted CB (*B* = 0.47, *p* < 0.001), and age emerged as a significant negative predictor (*B* = −0.33, *p* < 0.001). Overall, this model explained 26.1% of the variance in CB (*R*^2^ = 0.26, adjusted *R*^2^ = 0.26, *F*(3, 441) = 51.83, *p* < 0.001).

In the second model, COANT significantly predicted CB (*B* = 0.21, SE = 0.04, *β* = 0.26, *t* = 6.04, *p* < 0.001). Gender (*B* = 0.54, *p* < 0.001) and age (*B* = −0.31, *p* < 0.001) remained significant. This model accounted for 27.3% of the variance (*R*^2^ = 0.27, adjusted *R*^2^ = 0.27, *F*(3, 441) = 55.26, *p* < 0.001).

The third linear regression demonstrated that PRP was a robust predictor of CB, with higher role perception associated with more frequent communication (*B* = 0.55, SE = 0.04, *β* = 0.52, *t* = 14.28, *p* < 0.001). Gender (*B* = 0.32, *p* = 0.004) and age (*B* = −0.28, *p* < 0.001) remained significant, and this model explained substantially more variance than the ATA or COANT models (*R*^2^ = 0.46, adjusted *R*^2^ = 0.46, *F*(3, 441) = 126.20, *p* < 0.001).

#### 3.6.2 Making-up behaviour (MUB)

A second set of models examined whether the same predictors explained variation in making-up behaviour, defined as reconciliation tendencies toward the pet, that is, how owners respond when they feel they might have wronged their companion animal. In the first model, ATA significantly predicted more frequent making-up behaviours (*B* = 0.56, SE = 0.12, *β* = 0.21, *t* = 4.62, *p* < 0.001). Gender also contributed positively (*B* = 0.32, *p* = 0.03), whereas age was a significant negative predictor (*B* = −0.25, *p* < 0.001). This model explained 15.8% of the variance in MUB (*R*^2^ = 0.16, adjusted *R*^2^ = 0.15, *F*(3, 441) = 27.55, *p* < 0.001).

When COANT was entered as a predictor, it showed a stronger effect (*B* = 0.33, SE = 0.04, *β* = 0.39, *t* = 9.18, *p* < 0.001). This model accounted for 25.8% of the variance (*R*^2^ = 0.26, adjusted *R*^2^ = 0.25, *F*(3, 441) = 51.01, *p* < 0.001).

The final model revealed that PRP was the strongest predictor of MUB (*B* = 0.52, SE = 0.04, *β* = 0.48, *t* = 11.81, *p* < 0.001), suggesting that perceiving the pet as socially important corresponds to more behaviours oriented towards making up. Age remained a significant negative predictor (*B* = −0.20, *p* < 0.001), whereas gender was no longer significant (*B* = 0.17, *p* = 0.172). PRP explained the largest proportion of variance (*R*^2^ = 0.33, adjusted *R*^2^ = 0.33, *F*(3, 441) = 72.15, *p* < 0.001), outperforming both ATA and COANT.

#### 3.6.3 Perceived social support (SS)

A final set of regression models assessed the extent to which the three constructs predicted perceived social support from companion animals. When ATA was entered as the predictor, it emerged as a significant positive correlate of SS (*B* = 0.73, SE = 0.12, *β* = 0.28, *t* = 5.94, *p* < 0.001). Age also negatively predicted SS (*B* = −0.10, *p* = 0.025), whereas gender was not significant. This model accounted for 10.5% of the variance (*R*^2^ = 0.11, adjusted *R*^2^ = 0.10, *F*(3, 441) = 17.28, *p* < 0.001).

In contrast, COANT showed a stronger association with SS (*B* = 0.35, SE = 0.04, *β* = 0.42, *t* = 9.40, *p* < 0.001), explaining 19.5% of the variance (*R*^2^ = 0.20, adjusted *R*^2^ = 0.19, *F*(3, 441) = 25.59, *p* < 0.001). Gender showed only a marginal effect (*p* = 0.088), and age was not significant.

PRP was the strongest overall predictor of SS, with a substantial positive effect (*B* = 0.72, SE = 0.04, *β* = 0.67, *t* = 18.61, *p* < 0.001), indicating that individuals who ascribed more salient roles to their pets reported greater emotional support. Neither gender (*p* = 0.566) nor age (*p* = 0.410) contributed significantly in this model. PRP accounted for markedly more variance than either ATA or COANT (*R*^2^ = 0.46, adjusted *R*^2^ = 0.46, *F*(3, 441) = 124.53, *p* < 0.001).

### 3.7 Mediation analysis

Given that both ATA and COANT showed consistent associations with the three relational outcomes, mediation analyses were conducted to examine whether these links operated through PRP. PRP was included as a mediator on both conceptual and empirical grounds: beyond reflecting beliefs about the pet’s social position, it also showed the strongest direct effects on CB, MUB, and SS. Thus, PRP was conceptualised not as a competing predictor, but as a potential explanatory mechanism through which broader attitudes and anthropomorphic beliefs translate into concrete relational behaviours.

Mediation was evaluated using bootstrapped confidence intervals (5,000 resamples) to estimate indirect effects, providing robust inference under the non-normal distribution of the data.

#### 3.7.1 Communication behaviour (CB)

For CB, ATA showed a significant indirect effect through PRP (*B* = 0.33, SE = 0.06, *z* = 5.19, *p* < 0.001, 95% CI [0.20, 0.50]), while the direct effect remained significant (*B* = 0.26, SE = 0.09, *z* = 2.79, *p* = 0.005), indicating partial mediation. PRP was a strong predictor of CB (*B* = 0.52, SE = 0.06, *z* = 9.29, *p* < 0.001), and ATA significantly predicted PRP (B = 0.64, SE = 0.13, *z* = 5.11, *p* < 0.001). The model explained 47.1% of the variance in CB (*R*^2^ = 0.47).

For COANT, an indirect effect through PRP was also observed (*B* = 0.13, SE = 0.02, *z* = 6.22, *p* < 0.001, 95% CI [0.09, 0.17]). COANT additionally maintained a significant direct effect on CB (B = 0.08, SE = 0.03, *z* = 3.29, *p* = 0.001), again indicating partial mediation. PRP strongly predicted CB (B = 0.52, SE = 0.06, *z* = 8.99, *p* < 0.001), and COANT significantly predicted PRP (*B* = 0.25, SE = 0.03, *z* = 8.07, *p* < 0.001). This model explained 47.0% of the variance in CB (*R*^2^ = 0.47).

Gender positively predicted CB both directly and indirectly through PRP, whereas age showed both direct and indirect negative paths.

**Figure 1:**
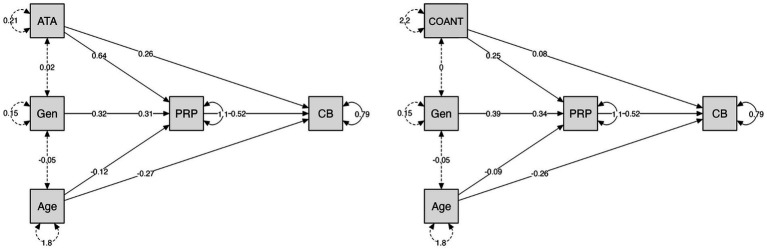
Comparison of mediation plots for communication behaviour (CB).

A visual summary of both mediation models is shown in Figure 1.

#### 3.7.2 Making-up behaviour (MUB)

The same mediation procedure was applied to MUB. ATA demonstrated a significant indirect effect through PRP (*B* = 0.31, SE = 0.06, *p* < 0.001, 95% CI [0.19, 0.47]), while its direct effect remained significant (*B* = 0.25, *p* = 0.034), indicating partial mediation. PRP strongly predicted MUB (*B* = 0.49, SE = 0.06, *z* = 7.84, *p* < 0.001), and ATA significantly predicted PRP (*B* = 0.64, *p* < 0.001). This model explained 33.7% of the variance in MUB (*R*^2^ = 0.34).

For COANT, an indirect pathway through PRP was also significant (*B* = 0.11, SE = 0.02, *p* < 0.001, 95% CI [0.08, 0.14]). A significant direct effect remained (*B* = 0.22, *p* < 0.001), consistent with partial mediation. COANT and PRP both significantly predicted MUB (*B* = 0.33 and *B* = 0.43, both *p* < 0.001), and COANT predicted PRP (*B* = 0.25, *p* < 0.001). This model accounted for slightly more variance (*R*^2^ = 0.39) than the ATA model.

In both models, gender showed full mediation through PRP, whereas age showed partial mediation.

**Figure 2:**
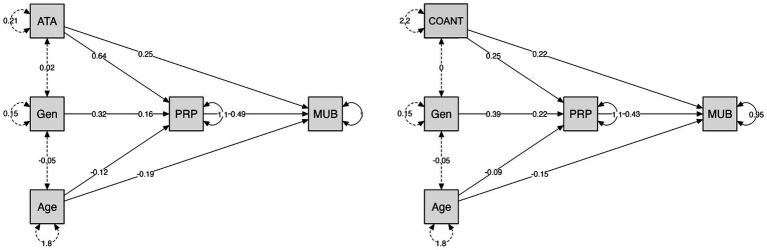
Comparison of mediation plots for making-up behaviour (MUB).

Visual representations of these pathways are shown in Figure 2.

#### 3.7.3 Perceived social support (SS)

For SS, ATA exerted a significant indirect effect via PRP (*B* = 0.44, SE = 0.08, *p* < 0.001, 95% CI [0.28, 0.63]), while the direct effect remained significant (*B* = 0.29, *p* = 0.003), indicating partial mediation. PRP strongly predicted SS (*B* = 0.69, SE = 0.05, *z* = 13.23, *p* < 0.001), and ATA significantly predicted PRP (*B* = 0.64, *p* < 0.001). The model explained 46.9% of the variance in SS (*R*^2^ = 0.47).

For COANT, mediation through PRP was also observed (*B* = 0.16, SE = 0.02, *p* < 0.001, 95% CI [0.12, 0.21]), with a significant remaining direct path (*B* = 0.19, *p* < 0.001). PRP predicted SS (*B* = 0.65, *p* < 0.001), and COANT predicted PRP (*B* = 0.25, *p* < 0.001). This model explained 50.0% of the variance in SS (*R*^2^ = 0.50).

Gender and age did not show direct effects on SS but demonstrated significant indirect effects via PRP, indicating full mediation for gender and partial mediation for age.

**Figure 3:**
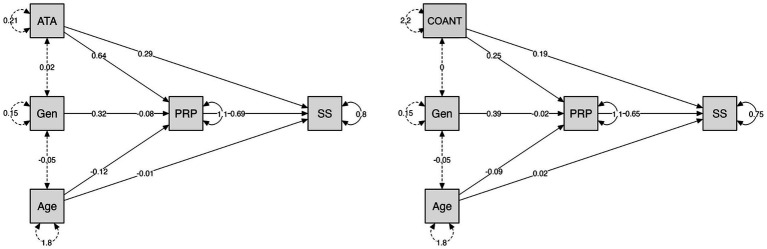
Comparison of mediation plots for social support (SS).

A visual comparison of the two mediation models is shown in Figure 3.

### 3.8 Moderated mediation by pet type

To examine whether the indirect effects through PRP differed between cat and dog owners, moderated mediation models were estimated using 5,000 bootstrapped resamples. Across all outcomes, pet type did not significantly moderate the pathways from ATA or COANT to PRP (all interaction terms *p* > 0.10), indicating that the mediating role of PRP was statistically comparable between species groups.

#### 3.8.1 Communication behaviour (CB)

Both predictors showed significant indirect effects on CB through PRP for cat and dog owners. For ATA, the indirect effect was significant for dog owners (*B* = 0.38, 95% CI [0.23, 0.54]) and for cat owners (*B* = 0.25, 95% CI [−0.00, 0.54]), although the interaction term was not significant (*p* = 0.294). Similarly, COANT demonstrated significant indirect effects for both groups (dogs: *B* = 0.16, 95% CI [0.12, 0.20]; cats: *B* = 0.14, 95% CI [0.07, 0.23]), with no significant moderation (*p* = 0.659).

#### 3.8.2 Making-up behaviour (MUB)

A comparable pattern emerged for MUB. ATA showed a significant indirect effect through PRP for both dog owners (*B* = 0.35, 95% CI [0.21, 0.53]) and cat owners (*B* = 0.23, 95% CI [0.04, 0.45]), despite a non-significant interaction term (*p* = 0.294). COANT also produced significant indirect effects for both dogs (*B* = 0.11, 95% CI [0.07, 0.16]) and cats (*B* = 0.10, 95% CI [0.05, 0.16]), and again pet type did not moderate these effects (*p* = 0.735). Thus, although indirect effects tended to be stronger among dog owners, the mediating mechanism was statistically similar across groups.

#### 3.8.3 Perceived social support (SS)

For SS, ATA showed significant indirect effects for dog owners (*B* = 0.50, 95% CI [0.32, 0.70]) and cat owners (*B* = 0.33, 95% CI [0.01, 0.67]), with no significant moderation (*p* = 0.294). COANT displayed the same pattern, with significant effects that were slightly larger for dog owners (*B* = 0.17, 95% CI [0.12, 0.22]) than for cat owners (*B* = 0.15, 95% CI [0.08, 0.24]), but the interaction term again was not significant (*p* = 0.735).

Overall, the moderation analyses showed that pet type did not significantly alter the indirect pathways from ATA or COANT to any relational outcome. Although conditional effects were descriptively stronger among dog owners, these differences were not statistically significant, indicating that the mediating function of PRP operates similarly for both species’ groups.

### 3.9 Anthropomorphisation valence

An exploratory analysis was conducted to examine the emotional valence of anthropomorphic expressions used by respondents when referring to their companion animals. Two single-item measures assessed distinct forms of anthropomorphism: positive anthropomorphism, defined as the use of affectionate, child-like terms (e.g., “my baby,” “kiddo”), and negative anthropomorphism, defined as the use of colloquial expressions implying mischievous or undesirable behaviour (e.g., “jerk,” “bastard”). Both items were rated on 7-point Likert scales ranging from never to always.

**Figure 4:**
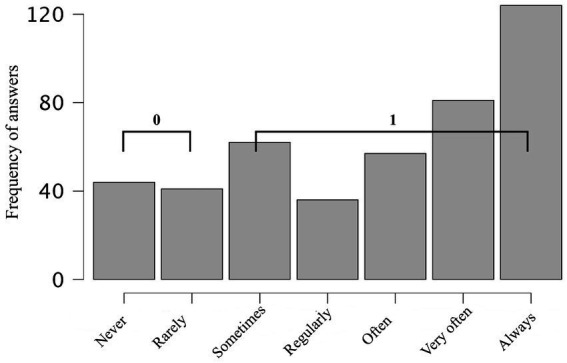
Data distribution for question: Do you use endearing words or expressions (e.g., “my baby,” “mummy’s baby,” “daddy’s baby,” or “kiddo”) when referring to ___?

**Figure 5:**
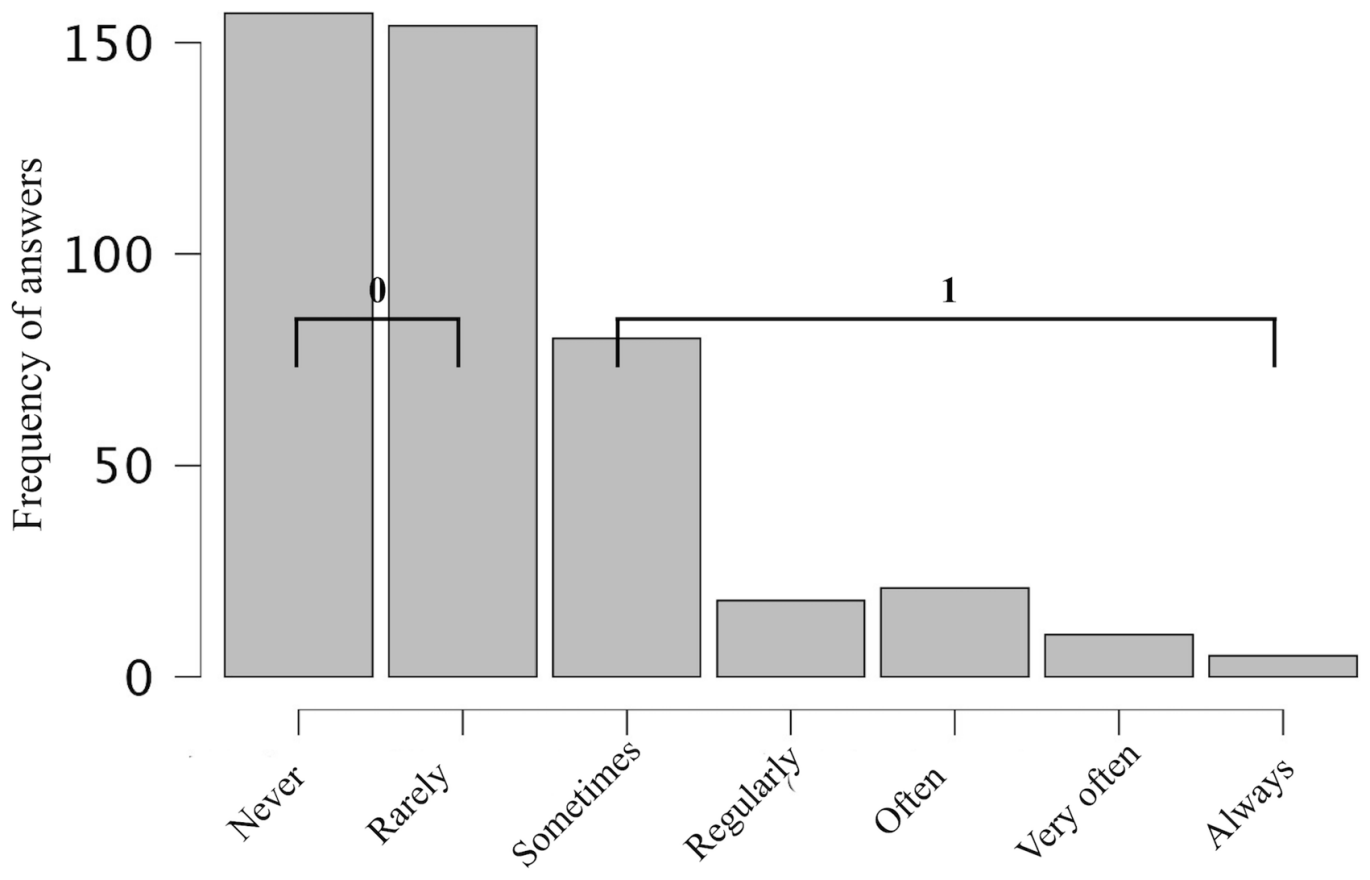
Data distribution for the question: Do you use uncomplimentary or reproachful words or expressions (e.g., “jerk,” or “bastard”) when referring to ___?

To facilitate interpretation and align with the empirical response distribution (Figures 4, 5), both variables were recoded into binary categories. Responses of never or rarely were coded as 0 (absence of behaviour), while all remaining responses (sometimes to always) were coded as 1 (presence of behaviour). This approach follows established practices in behavioural research (e.g., Daly et al., 2016) and supports clearer contingency-table comparisons (Table 5).

**Table 5:** Contingency analysis for anthropomorphic direction.

		Negative item		
Positive item		No	Yes	Total
No	Count	68	17	85
	% of total	15.3%	3.8%	19.1%
Yes	Count	243	117	360
	% of total	54.6%	26.3%	80.9%
Total	Count	311	134	445
	% of total	69.9%	30.1%	100%

A chi-squared test of independence revealed a significant association between positive and negative anthropomorphism, *χ*^2^(1, *N* = 445) = 5.11, *p* = 0.024, indicating that the two tendencies were not statistically independent. The effect size was small (Phi = 0.11; Cramér’s *V* = 0.11). The majority of respondents (80.9%) reported engaging in positive anthropomorphism, whereas fewer endorsed negative anthropomorphism (30.1%). Importantly, most individuals who used negative anthropomorphism also used positive anthropomorphism (117 of 134), whereas positive-only anthropomorphism was far more common (54.6%) than negative-only anthropomorphism (3.82%). These patterns suggest that valenced anthropomorphic expressions are asymmetrically distributed, with negative anthropomorphism emerging mainly as a subset within a broader affectionate repertoire.

To examine potential demographic correlates of valenced anthropomorphism, two binary variables were created to isolate respondents who anthropomorphised only positively or only negatively. Chi-squared analyses showed that gender was significantly associated with positive-only anthropomorphism, *χ*^2^(1, *N* = 445) = 5.19, *p* = 0.023, with women more likely than men to use exclusively positive, endearing terms. No association emerged between gender and negative-only anthropomorphism, *χ*^2^(1, *N* = 445) = 0.28, *p* = 0.599.

Education level showed a marginal association with positive-only anthropomorphism when considering the likelihood ratio statistic, *χ*^2^(3, *N* = 445) = 8.11, *p* = 0.044, although the Pearson chi-square was non-significant, *χ*^2^(3, *N* = 445) = 6.22, *p* = 0.101. Descriptively, participants with primary or middle-school education were less likely to anthropomorphise positively, whereas those with higher education showed higher endorsement. No association was found between education level and negative-only anthropomorphism, *χ*^2^(3, *N* = 445) = 2.65, *p* = 0.449.

Additional chi-squared tests examined age, spirituality, professional involvement with animals, pet species, pet sex, pet age, and duration of ownership. None of these variables was significantly related to either positive-only or negative-only anthropomorphism, suggesting that the direction of anthropomorphism may be influenced by factors not captured in the present dataset (e.g., attachment style, emotional regulation tendencies, or culturally shaped caregiving norms).

To explore whether anthropomorphic valence corresponded to broader relational tendencies, a series of Mann–Whitney *U* tests was conducted comparing positive-only and negative-only anthropomorphisers to all other respondents across PRP, CB, MUB, SS, ATA, and COANT.

Participants who engaged in positive-only anthropomorphism reported significantly higher PRP, CB, MUB, and SS (*U* = 5,111–5,374, all *p* < 0.05), with rank-biserial correlations ranging from 0.34 to 0.48, indicating small-to-moderate effects. No significant differences emerged for ATA or COANT. In contrast, participants who anthropomorphised only negatively scored significantly lower on PRP, CB, MUB, and SS (*U* = 17,609–20,458, all *p* < 0.05), with rank-biserial correlations between −0.17 and −0.28. Differences in COANT approached significance, whereas ATA did not differ significantly. These findings suggest that the valence of anthropomorphic expression reflects meaningful qualitative distinctions: positively valenced anthropomorphism aligns with stronger relational engagement and perceived support, whereas negatively valenced anthropomorphism corresponds to weaker relational indicators.

Overall, this exploratory analysis highlights that anthropomorphic valence is not uniformly expressed nor demographically determined. Instead, positively and negatively valenced anthropomorphism seems to follow distinct relational patterns, offering insight into the different ways owners perceive and emotionally relate to their companion animals.

## 4 Discussion

The present study examined how attitudes toward animals, comparative anthropomorphism, and beliefs about the pet’s social role jointly shape the behavioural and emotional dynamics of the relationships between animal guardians and their pets in Romania. Across analyses, pet role perception (PRP) emerged as the central explanatory mechanism connecting owners’ beliefs with their communicative, reconciliatory, and socio-emotional behaviours. This supports the notion that the social meaning attributed to pets plays a decisive role in shaping human-animal interactions. In addition to these primary associations, the study also included two exploratory questions on anthropomorphic valence. These questions examined whether owners’ relational actions were significantly correlated with the emotional tone of anthropomorphic attributions (positive, negative, or mixed). According to our initial findings, anthropomorphism based on emotional attribution might represent a unique dimension of owner-pet interaction, which calls for more conceptual and empirical investigation.

To clarify the overarching framework of this study, it is useful to revisit the working definitions of the key constructs and to situate them in relation to one another. Attitudes toward animals (ATA) reflect broad moral-affective orientations toward animals as a category, capturing general beliefs about animals’ emotional capacities, cognitive abilities, and moral standing. Comparative anthropomorphisation (COANT) represents a more situational and relational process, reflecting owners’ cognitive judgments about the similarity between human and animal mental and emotional capacities. Pet role perception (PRP) describes how these broader orientations and comparative appraisals are translated into socially meaningful roles assigned to the pet within the household (e.g., family member, child, friend). Finally, anthropomorphic valence refers to the emotional tone accompanying anthropomorphic attribution, distinguishing between positive, negative, or mixed affective framings of the pet. Within the proposed framework, general attitudes toward animals provide a distal evaluative context, comparative anthropomorphism supplies a proximal cognitive basis for role assignment, pet role perception functions as a relational concept shaping everyday interaction, and anthropomorphic valence modulates the affective quality through which these relationships are enacted. This integrative perspective highlights the multi-level nature of anthropomorphic processes in human-animal relationships and situates the empirical findings within a coherent theoretical architecture.

### 4.1 Attitudes, anthropomorphism, and the formation of pet role perception

Both attitudes toward animals (ATA) and comparative anthropomorphisation (COANT) predicted the perceived role of the pet (PRP), but COANT showed the strongest and most consistent associations, suggesting that comparative anthropomorphism operates as a more proximal cognitive determinant of role assignment. This result highlights the importance of perceived human-animal similarity in how owners place their pets within the social hierarchy of their homes. These results are consistent with the framework proposed by Bouma et al. (2023), who also found that anthropomorphism and pet role perception were key predictors of owner-pet relational dynamics. In contrast to Bouma et al. (2023), who compared COANT with a general anthropomorphism measure (IDAQ), our study introduced ATA to examine whether moral-affective orientations are specifically responsible for PRP and relational behaviours.

In this study, ATA, which was intended to capture broad moral and affective orientations toward animals, showed weaker and less consistent associations with pet role perception and relational outcomes. This pattern should be interpreted with caution, given the low internal reliability (*α* = 0.38) of the ATA subscale in the present sample. Accordingly, findings involving ATA should be considered exploratory. Reduced internal consistency likely attenuated observable effects and suggests that the subscale may encompass heterogeneous items combining emotional, cognitive, and moral considerations, which may have reduced its internal coherence in this sample, reinforcing the interpretation that COANT functions as a more proximal determinant of role assignment than broader evaluative attitudes toward animals. Future research would benefit from refining this construct or employing alternative, more homogeneous measures of animal-related attitudes when examining their role in human-animal relational processes.

Beyond replicating Bouma et al.’s findings in an understudied cultural context, our results extend their relational model by demonstrating that PRP retains its central predictive role within a Romanian population. Our findings of similar associations suggest that these relational mechanisms may be relatively stable across different companion-animal environments and cultural settings.

The distinction between ATA and COANT supports the conceptual argument that attitudes toward animals reflect general evaluative orientations. In contrast, comparative anthropomorphism captures situational, relational attributions that more directly inform role assignment and perception. This differentiation is consistent with broader theoretical perspectives describing anthropomorphism as an interpretative tool for navigating social interactions with non-human partners. In this sense, anthropomorphism may function as a cognitive framework through which owners organise expectations, interpret behaviour, and construct relational meaning.

### 4.2 Pet role perception as a relational mechanism

Across all relational outcomes investigated in our study (communication behaviour, making-up behaviour, and perceived social support), the mediation patterns consistently indicated that pet role perception functions as a key relational mechanism through which attitudes and anthropomorphic interpretations are translated into everyday interaction. Owners who viewed their pets as emotionally significant household members reported more frequent affiliative behaviours, greater efforts to repair relational ruptures, and stronger perceptions of emotional support.

Notably, the strength of the mediation pathways was highly consistent across species. Although dog owners scored higher on comparative anthropomorphism and social support in group-level comparisons, the moderated mediation analyses indicated that the psychological processes linking attitudes, anthropomorphism, and relational behaviour were statistically equivalent for cat and dog owners. This suggests that PRP operates as a species-general relational mechanism, rather than one that is specific to companion animals. These findings suggest that once a pet is granted a salient social role, the relational dynamics that follow appear to function comparably across species. This indicates that species-based distinctions may become secondary once a companion animal is integrated into the household through a meaningful relational role.

### 4.3 Anthropomorphic valence as an affective dimension of owner-pet relationships

The exploratory analysis of anthropomorphic valence provides an additional layer of insight into how owners emotionally interpret and relate to their pets. While anthropomorphism is often treated as a unitary construct reflecting the degree to which human-like qualities are attributed to animals, the present findings demonstrate that the emotional tone of these attributions differentiates relational patterns in meaningful ways. Most respondents engaged in positively valenced anthropomorphism, whereas negative-only anthropomorphism was rare but present in the study sample. Importantly, these two forms were not mutually exclusive: individuals who used negative anthropomorphic terms almost always used positive ones as well, suggesting that negative language tends to emerge within a broader affectionate repertoire rather than as a standalone communicative style. Additionally, the negatively framed item did not capture whether words such as “jerk” or “bastard” were employed ironically or only in response to behaviours the owner considered problematic. This limits interpretability, as anthropomorphic valence cannot be inferred from wording alone, and context should be taken into consideration.

Patterns of relational behaviour also mirrored the asymmetry found in the use of anthropomorphic attribution. Participants who engaged exclusively in positive anthropomorphism reported substantially higher levels of pet role perception, communication behaviour and making-up behaviour. They also reported higher levels of perceived social support. Conversely, those who used only negatively valenced anthropomorphic expressions showed consistently lower scores across the same relational domains. These differences, though exploratory, indicate that anthropomorphic valence may reflect an affective orientation that shapes how people engage with their pets, influencing both behavioural tendencies and perceived relational closeness. Notably, valence effects were not reducible to general attitudes or to comparative anthropomorphism, neither of which differed meaningfully between positive-only and negative-only groups. This suggests that emotional tone captures a distinct dimension of owner-pet interaction, one that is not yet adequately represented in existing anthropomorphism scales.

Although the mechanisms underlying valenced anthropomorphism cannot be fully determined from the present data, several interpretative possibilities arise. Positive anthropomorphism may function as a relational resource, reinforcing intimacy, emotional expressiveness, and affiliative scripts within the owner-pet dyad. In contrast, negative anthropomorphism may reflect frustration, unmet behavioural expectations, or the projection of human social norms onto animal behaviour, potentially resulting in relational distance or reduced perceived support. The absence of demographic predictors for negative-only anthropomorphism further suggests that these patterns may be shaped by psychological or situational factors that were not assessed here (e.g., attachment style, emotion regulation tendencies, cultural norms of caregiving). Future research should investigate these possibilities, as valence may be an important differentiator of how anthropomorphism functions within everyday interactions.

Together, these findings indicate that anthropomorphic valence is not merely an anecdotal linguistic habit but may represent an affectively laden dimension of human-animal relationships that warrants systematic measurement. Integrating valence into theoretical models of anthropomorphism could improve our understanding of how owners interpret animal behaviour, assign relational meaning, and experience emotional support.

Additionally, the present findings suggest that anthropomorphic thinking functions as part of a broader relational process through which companion animals are construed as thinking and feeling partners rather than as passive objects, allowing accumulated interactional experience to shape owners’ future behavioural expectations and emotional responses, underscoring relationships as the appropriate level of analysis (Kieson, 2025) rather than a focus on individual actors.

### 4.4 Demographic correlates of anthropomorphism and relational behaviour

The present findings also revealed meaningful demographic patterns that parallel broader international trends. Younger respondents reported higher levels of anthropomorphism, pet role perception, and relational behaviours. Gender differences were likewise evident: women scored higher on several relational indicators, including communication behaviour and positive anthropomorphism. Although these demographic variables were not central predictors in the mediation models, they appear to shape the broader relational climate in which owners attach meaning to their pets.

### 4.5 Limitations and directions for future research

Several limitations should be considered when interpreting these findings. First, the use of convenience sampling restricts the generalisability of the results to the broader population of Romanian pet owners. It is important to specify that, based on previous experiences with data collection from respondents in Romania in the human-animal interaction domain, the convenience sampling method appears to be the most efficient one (Rusu et al., 2019). Second, the ATA subscale demonstrated notably low internal reliability in this sample, which may have attenuated its predictive power and suggests that the construct may operate differently across cultural contexts or may encompass multiple conceptual domains. Third, the study relied exclusively on self-reported data, introducing the possibility of social desirability biases or inaccuracies in owners’ perceptions of their own behaviours.

A further limitation concerns the operationalisation of anthropomorphic valence. Although the single-item indicators provided an initial window into the emotional tone of anthropomorphic attribution, this aspect of the study should be considered exploratory in nature. Single-item measures necessarily constrain reliability and limit the depth of inference that can be drawn regarding affective attribution. As such, conclusions concerning anthropomorphic valence should be interpreted as preliminary. Future research should prioritise the development and validation of multi-item measures capable of capturing the complexity, contextual variability, and affective nuance of anthropomorphic expressions, thereby enabling more robust tests of how emotional framing shapes human-animal relational processes. Additionally, observational, experimental, or longitudinal designs may offer more fine-grained insight into how anthropomorphic attribution and role perception dynamically unfold in everyday owner-pet interactions, allowing for stronger causal inference.

### 4.6 Practical implications

The present findings have several practical implications not only for the category of pet owners, but also for professionals working with companion animals, particularly in veterinary, behavioural, and adoption-support contexts, as well as for social workers and psychologists addressing various aspects of human-animal interactions. First, the central role of pet role perception (PRP) suggests that the way owners conceptualize their pets (as children, friends, full family members, or just animals around their household) shapes not only their day-to-day interactions but also their expectations of professional services. Veterinary teams, for example, may benefit from assessing owners’ relational schemas when discussing treatment plans, behavioural issues, or end-of-life decisions. Owners who assign highly human-like roles to their pets may require different communication strategies, particularly when navigating emotionally charged situations where anthropomorphic beliefs influence decision-making. In the context of applied psychology and animal welfare, our findings align with research on pet attachment among Romanian pet owners (Rusu et al., 2019), which demonstrated individual differences in human-animal rapport moderated by attachment style. Specifically, Rusu et al. (2019) found that anxiously attached individuals exhibited greater empathy toward animals and higher levels of pet anthropomorphisation, whereas avoidantly attached individuals displayed reduced empathy and lower anthropomorphisation tendencies. Consequently, human-animal attachment represents a critical variable warranting investigation in future studies aimed at fostering secure, welfare-promoting human-companion animal relationships.

Second, the strong associations between PRP and relational behaviours such as communication, reconciliation, and perceived support highlight the potential value of educational interventions that address owners’ expectations and interpretations of pet behaviour. Misaligned anthropomorphic assumptions (especially when negatively valenced) may increase frustration or misunderstanding, potentially leading to welfare challenges or breakdowns in the owner-pet relationship. Adoption and fostering programs could integrate brief assessments or guided discussions on anthropomorphic tendencies and role expectations to promote more stable and resilient human-animal bonds.

Finally, the exploratory findings on anthropomorphic valence suggest that the emotional tone of owners’ attributions may serve as an indicator of relational quality. Positively valenced anthropomorphism was associated with stronger relational engagement, while negative-only anthropomorphism corresponded to weaker affiliative patterns. These differences may be relevant in early identification of at-risk owner-pet relationships, including those vulnerable to relinquishment. Tailored counselling or behavioural support could be directed toward owners whose emotional attributions reveal relational strain, potentially mitigating escalating conflict or abandonment risk.

## 5 Conclusion

Our findings demonstrate that attitudes toward animals and comparative anthropomorphism shape human-pet relationships through the social roles assigned to companion animals. The present study reinforces the view that anthropomorphism is not merely a cognitive distortion or source of interpretative error but a complex psychological mechanism that can structure, enrich, and at times challenge human-animal relationships. By showing that comparative anthropomorphism is a strong and proximal predictor of how owners view their pets’ social roles, and that anthropomorphic valence further differentiates relational patterns, this study highlights that anthropomorphism operates not only in degree but also in affective quality.

## Data Availability

The raw data supporting the conclusions of this article will be made available by the authors, without undue reservation.
